# Ossification and increased bone mineral density with zoledronic acid in a patient with lung adenocarcinoma: A case report

**DOI:** 10.3892/etm.2014.1914

**Published:** 2014-08-18

**Authors:** KOICHI KURISHIMA, GEN OHARA, KATSUNORI KAGOHASHI, NORIO TAKAYASHIKI, TOMOHIRO TAMURA, TOSHIHIRO SHIOZAWA, KUNIHIKO MIYAZAKI, MIO KAWAGUCHI, HIROAKI SATOH, NOBUYUKI HIZAWA

**Affiliations:** 1 Division of Respiratory Medicine, Mito Medical Center, University of Tsukuba, Mito, Ibaraki 310-0015, Japan; 2 Division of Pathology, Mito Medical Center, University of Tsukuba, Mito, Ibaraki 310-0015, Japan; 3 Division of Respiratory Medicine, Faculty of Medicine, University of Tsukuba, Tsukuba, Ibaraki 305-8575, Japan

**Keywords:** bone metastasis, zoledronic acid, lung cancer, bone mineral density, ossification

## Abstract

Cases of ossification and increased bone mineral density (BMD) at sites of bone metastasis following zoledronic acid (ZA) treatment have not been reported. The current study presents the case of a 65-year-old patient with lung adenocarcinoma and bone metastases in the lumbar vertebrae and femurs. Ossification and an increase in BMD at the metastatic sites was achieved following treatment with ZA and irradiation of the bone metastatic sites. The patient was able to maintain a normal lifestyle for over two years, despite the bone metastases. Therefore, as treatment with ZA was demonstrated to improve patient quality of life, physicians should consider this treatment strategy, particularly for the treatment of metastasis in weight-bearing bones.

## Introduction

Lumbar vertebrae and femur bone metastases are associated with an increased risk of fracture and a decreased quality of life for the affected individual (1,2). Zoledronic acid (ZA) is understood to prevent these risks by inhibiting the function of osteoclast cells; thus, reducing bone loss (3). Application of ZA and irradiation to the bone metastases have been hypothesized to facilitate ossification and cause an increase in bone mineral density (BMD); however, to the best of our knowledge, these phenomena have not yet been reported. In addition to conventional irradiation, novel treatments, including systemic chemotherapy and molecular target agents, such as erlotinib and bervacizumab, have represented a significant advance in lung adenocarcinoma treatment (4,5). These novel treatment strategies combined with ZA are now recommended for the treatment of the majority of patients with lung adenocarcinoma with bone metastasis (3,6). The current study presents the case of a patient with lung adenocarcinoma who exhibited an increased BMD in the metastatic sites following ZA administration and irradiation. An increase in ossification was also observed. The patient was able to maintain a normal lifestyle for more than two years, despite the lumbar vertebrae and femur bone metastases. Written informed consent was obtained from the patient’s family.

## Case report

**Figure 1:**
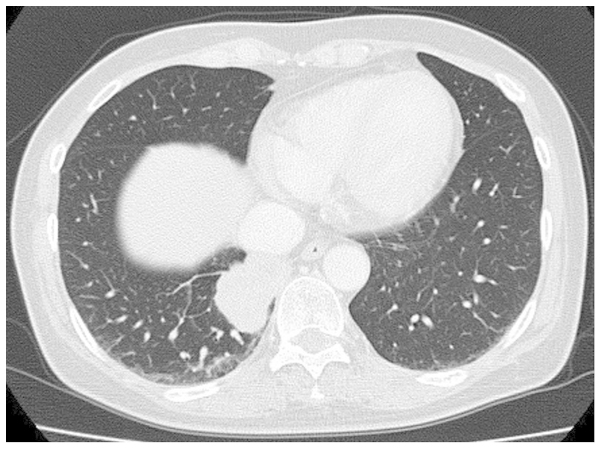
Chest computed tomography scan at the time of first admission showing a large mass in the right lower lobe of the lungs.

**Figure 2:**
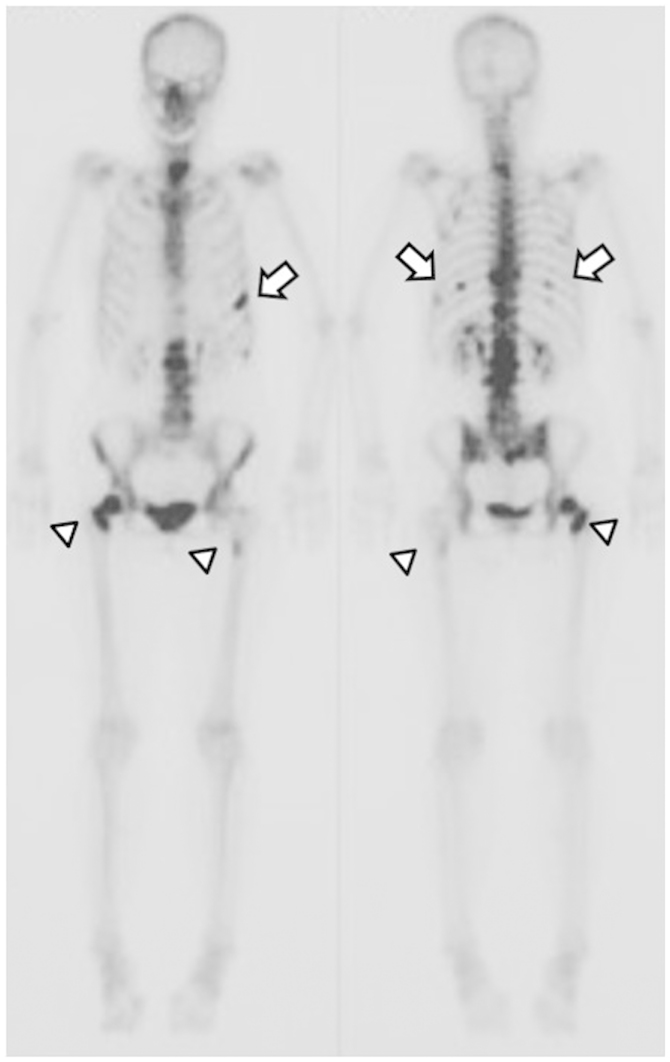
Bone scan at the time of first admission showing a marked accumulation of ^99m^Tc in the vertebrae (C7, Th9, Th11, L1 and L2), femurs (arrow head) and ribs, as indicated by the arrows.

A 63-year-old female with no history of smoking was admitted to Mito Medical Center, University of Tsukuba-Mito Kyodo General Hospital (Mito, Japan) after presenting with lumbago and pain around the hip joints for five months. At admission, chest radiography and computed tomography (CT) scans revealed a 3.5×2.5 cm mass in the lower right lobe of the lungs, with multiple intrapulmonary metastases (Fig. 1). Bone scans demonstrated marked accumulation of ^99m^Tc in the vertebrae (C7, Th9, Th11, L1 and L2), femurs and ribs (Fig. 2). In addition, an abdominal CT scan revealed a metastatic nodule in the liver. Histopathological specimens, which were obtained bronchoscopically from the mass in the right lung, revealed an adenocarcinoma with an exon 19 deletion in the epidermal growth factor receptor (EGFR) gene. The patient received a total hip replacement due to a right femoral neck fracture. Subsequently, irradiation was applied to the metastatic sites in the lumbar vertebrae and femurs, and four courses of chemotherapy with carboplatin, pemetrexed and bevacizumab were administered. The patient received 4 mg ZA every month for 24 months. The response to the chemotherapy was evaluated as a partial response; thereafter, the patient received 20 courses of maintenance chemotherapy with pemetrexed and bevacizumab.

**Figure 3:**
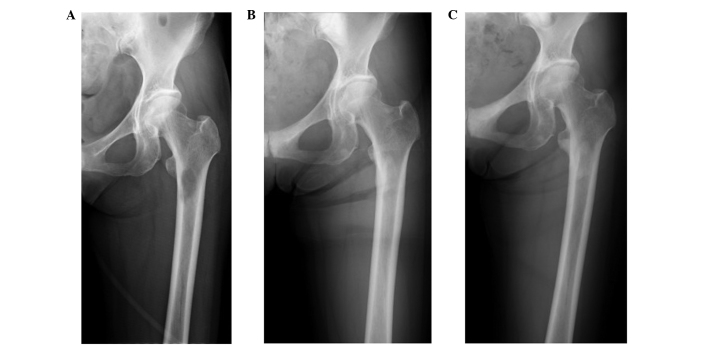
Plain radiography images showing changes in the metastatic site of the left femoral neck at (A) pretreatment, (B) 13 months following ZA initiation and (C) 21 months following ZA initiation. ZA, zoledronic acid.

Ossification of the metastatic site in the left femoral neck was observed using plain radiography three months following the initiation of ZA treatment (Fig. 3). The BMD was measured by dual-energy X-ray absorptiometry (DEXA; DPX-Bravo; GE Healthcare, Tokyo Japan). Table I shows the change in the BMD prior to, at 8 months and at 20 months following the initiation of ZA administration. An increased BMD was observed in the metastatic sites (L1, L2 and left femur), as well as the non-metastatic sites (L3 and L4). The patient did not undergo any adverse events following ZA treatment, including biphosphonate-associated osteonecrosis of the jaw (BRONJ), fever or renal toxicity. Following recurrence in the primary lesion and liver metastasis, the patient received gefitinib therapy. The patient survived with no evidence of bone metastasis in other sites for two years. However, the patient had liver and pulmonary recurrence and succumbed to the disease three years following the diagnosis of lung adenocarcinoma.

## Discussion

ZA is a third-generation nitrogen-containing parenteral bisphosphonate prescribed for the treatment of bone metastases caused by solid tumors (7). In patients with lung cancer, ZA exhibits a favorable protective effect against skeletal-associated events, including bone fracture and severe pain requiring irradiation and opioids (7). Although ZA has a well-established tolerability profile and may be administered safely as a long-term therapy, preventive measures are required to avoid a number of severe side effects, including BRONJ and renal toxicity, which have been observed in a small number of patients receiving long-term therapy (8). BRONJ is characterized by the unexpected appearance of necrotic bone in the oral cavity (8). In the current patient, cyclic administration of intravenous ZA resulted in ossification and an increase in the BMD of the lumbar spine and femur bone. The observations of the present study revealed that the use of ZA was associated with a clinical and radiological benefit. The patient also underwent irradiation therapy on the metastatic sites, platinum-based chemotherapy and gefitinib therapy. Apparent ossification with an improvement in the BMD was observed following the completion of irradiation, and the treatment was continued with ZA and systemic chemotherapy. Although the most effective treatment strategies for metastases remain unknown, it was hypothesized that ZA had an important effect on a favorable outcome without any adverse side effects, including BRONJ. Bone metastasis has been identified as one of the unfavorable prognostic factors in patients with lung cancer (9). However, in the present study, the patient was found to have a mutation in the EGFR gene, which was evaluated as a favorable prognostic factor. Long-term survivors with bone metastasis have rarely been reported (10), and the incidence rate of complete remission from bone metastasis in patients with non-small-cell lung carcinoma (NSCLC) is low (11). Although currently rare, treatment with ZA for lung cancer patients with bone metastasis, particularly for those that are indicative for other intensive therapies, including the patient in the present study, should be considered for future cases.

There have been a few reported cases of ossification at the metastatic site following administration of ZA (12). Kawai *et al* reported a patient with large cell lung cancer who received ZA treatment and subsequently exhibited marked regression of the thoracic vertebrae following irradiation applied to the metastatic site (12). Ikeda *et al* also presented two cases of patients with NSCLC who were successfully treated with irradiation applied to the metastatic sites, systemic chemotherapy and ZA (13). Ossification in the thoracic spine of one patient and in the rib of the other patient were observed (13). The BMD, as measured by DEXA, increases with the administration of ZA in post-menopausal osteoporosis patients (14), pediatric patients with spinal cord injury (15) and children with type III osteogenesis imperfecta (16). However, to the best of our knowledge, no study has revealed an improved BMD in the metastatic sites following the administration of ZA.

In conclusion, the results of the present study indicated that ZA enhanced ossification in the metastatic sites. In addition, an increase in the BMD occurred as a result of the formation of the metastatic bone lesions by irradiation, systemic chemotherapy and ZA. As the patient was able to maintain a normal lifestyle following treatment, the new bone appeared to have similar mechanical properties to that of normal non-metastatic bone. However, whether the increase in BMD with ossification in metastatic sites depends only on the efficacy of ZA remains inconclusive. Nonetheless, therapies that include ZA have been shown to improve the quality of life of patients; thus, should be considered for the treatment of bone metastasis, whilst considering potential side effects, such as BRONJ.

## Figures and Tables

**Table I:** Change in the BMD prior to and following the initiation of treatment with ZA.

	BMD (g/cm^2^)		
	
Site	Pretreatment	8-months from ZA initiation	20-months from ZA initiation
Lumbar spine			
L1	0.659	1.260	1.363
L2	0.654	1.706	1.970
L3	0.655	1.029	1.231
L4	0.767	0.917	0.949
Neck of left femur	0.772	0.779	0.849

BMD, bone mineral density; ZA, zoledronic acid.
